# The Role of Mitochondrial Impairment and Oxidative Stress in the Pathogenesis of Lithium-Induced Reproductive Toxicity in Male Mice

**DOI:** 10.3389/fvets.2021.603262

**Published:** 2021-03-24

**Authors:** Mohammad Mehdi Ommati, Mohammad Reza Arabnezhad, Omid Farshad, Akram Jamshidzadeh, Hossein Niknahad, Socorro Retana-Marquez, Zhipeng Jia, Mohammad Hassan Nateghahmadi, Khadijeh Mousavi, Aysooda Arazi, Mohammad Reza Azmoon, Negar Azarpira, Reza Heidari

**Affiliations:** ^1^Department of Bioinformatics, College of Life Sciences, Shanxi Agricultural University, Taigu, China; ^2^Department of Toxicology and Pharmacology, School of Pharmacy, Kerman University of Medical Sciences, Kerman, Iran; ^3^Pharmaceutical Sciences Research Center, Shiraz University of Medical Sciences, Shiraz, Iran; ^4^Department of Toxicology and Pharmacology, School of Pharmacy, Shiraz University of Medical Sciences, Shiraz, Iran; ^5^Department of Biology and Reproduction, Autonomous Metropolitan University, Mexico City, Mexico; ^6^College of Animal Science and Veterinary Medicine, Shanxi Agricultural University, Taigu, China; ^7^Transplant Research Center, Shiraz University of Medical Sciences, Shiraz, Iran

**Keywords:** antipsychotic drugs, bipolar disease, energy crisis, heavy metals, infertility, sperm

## Abstract

Lithium (Li^+^) is prescribed against a wide range of neurological disorders. Besides its excellent therapeutic properties, there are several adverse effects associated with Li^+^. The impact of Li^+^ on renal function and diabetes insipidus is the most common adverse effect of this drug. On the other hand, infertility and decreased libido is another complication associated with Li^+^. It has been found that sperm indices of functionality, as well as libido, is significantly reduced in Li^+^-treated men. These adverse effects might lead to drug incompliance and the cessation of drug therapy. Hence, the main aims of the current study were to illustrate the mechanisms of adverse effects of Li^+^ on the testis tissue, spermatogenesis process, and hormonal changes in two experimental models. In the *in vitro* experiments, Leydig cells (LCs) were isolated from healthy mice, cultured, and exposed to increasing concentrations of Li^+^ (0, 10, 50, and 100 ppm). In the *in vivo* section of the current study, mice were treated with Li^+^ (0, 10, 50, and 100 ppm, in drinking water) for five consecutive weeks. Testis and sperm samples were collected and assessed. A significant sign of cytotoxicity (LDH release and MTT assay), along with disrupted testosterone biosynthesis, impaired mitochondrial indices (ATP level and mitochondrial depolarization), and increased biomarkers of oxidative stress were detected in LCs exposed to Li^+^. On the other hand, a significant increase in serum and testis Li^+^ levels were detected in drug-treated mice. Moreover, ROS formation, LPO, protein carbonylation, and increased oxidized glutathione (GSSG) were detected in both testis tissue and sperm specimens of Li^+^-treated mice. Several sperm anomalies were also detected in Li^+^-treated animals. On the other hand, sperm mitochondrial indices (mitochondrial dehydrogenases activity and ATP levels) were significantly decreased in drug-treated groups where mitochondrial depolarization was increased dose-dependently. Altogether, these data mention oxidative stress and mitochondrial impairment as pivotal mechanisms involved in Li^+^-induced reproductive toxicity. Therefore, based on our previous publications in this area, therapeutic options, including compounds with high antioxidant properties that target these points might find a clinical value in ameliorating Li^+^-induced adverse effects on the male reproductive system.

## Introduction

Lithium (Li^+^) is used for the management of a wide range of neurological diseases (1). On the other hand, humans are also exposed to Li^+^ through different routes such as drinking water, foods, or environmental exposure (e.g., in miners or Li^+^-battery manufacturers) (2, 3). Nielsen (1998) has calculated that the dietary needs of Li^+^ are generally <0.05 mg/kg of feed/day for laboratory animals (4). In humans, Schrauzer (2002) has reported that 0.5–3 mg of Li^+^/day are needed (5). On the other hand, Aral and Vecchio-Sadus (2008) have reviewed that up to 10 mg/L of Li^+^ in serum is recommended to bipolar patients (2). They have also shown that the higher doses (10, 15, and 20 mg/L in blood) cause a moderate poisoning (10 mg/L), slurred speech and confusion (15 mg/L), and high fatality rate (20 mg/L) (2).

Even though many investigations reported that low levels of Li^+^ are precious in mitigating signs of bipolar disorders and depression (6–8) found that prolonged exposure to therapeutic doses of Li^+^ triggers complications among hospitalized patients (8). Hence, toxicity in the renal system (9–11), nervous system (12), thyroid glands (goiter) (13), and dermatological-related complications (14, 15), as well as circulatory system (16) under Li^+^ exposure have been earlier reported. On the other hand, it has been shown that this light metal can pass across the placental barriers and then induce teratogenic effects (17, 18). Besides the above-mentioned alterations, sterility is a well-described adverse phenomenon in Li^+^-treated groups.

Several cases of reproductive abnormalities have been reported in association with Li^+^ administration (16, 19–21). The first reproductive studies indicated that exposure to Li^+^ could trigger sexual anomalies in men (22, 23). In rats, Li^+^ induced a considerable decrement in steroidogenesis-related essential genes expression and then steroidogenesis and spermatogenesis impairment (24, 25); whereas, its deficiency can also mitigate the mammalian reproductive performance. In a prolonged exposure investigation, subfertility induced by reproductive toxicity was observed in male rats exposed orally to Li^+^ (26). Halder et al. (27) have also highlighted the pivotal roles of Li^+^ on the fine-tuning regulation of maturation in epididymal spermatozoa (27). Li^+^ chloride- challenged birds also showed significant degenerative alterations in germ cells and the epithelium of seminiferous tubules (28, 29). Acute exposure of albino rats to various doses of Li^+^ (200 and 400 μg/100 g body live weight; for 3 weeks) could cause a considerable decrement in the spermatogenesis, and the serum concentrations of prolactin, testosterone (T), luteinizing hormone (LH), and follicle-stimulating hormone (FSH), along with remarkable mitigation in the activities of steroidogenesis- related genes (3ß-HSD and 17ß -HSD); none of the above indices were influenced with a low dose (100 μg/100 g body live weight) of Li^+^ (30).

Oxidative stress (OS) and its associated adverse events are the most prevalent mechanisms for xenobiotics-induced adverse effects (31–33). The role of OS in the mechanism of metals-induced cytotoxicity has been widely investigated (34–36). There are several lines of evidence mentioning the role of OS in the pathogenesis of Li^+^-induced adverse effects in the testis tissue and semen in both experimental models and human cases (19, 20). For instance, many anomalies in the functionality of spermatozoa are attributed to the increased level of ROS in testis and semen (37–40) through severe impairments of DNA, interruption of plasma membrane integrity, and denaturation of proteins (41–43). However, there is no precise source for reactive oxygen species (ROS) and the induction of OS in the reproductive system of Li^+^-treated patients.

It has been found that mitochondria could be a critical target for Li^+^ adverse effects in biological systems (21, 44, 45). Li^+^ can adversely affect mitochondrial function in other tissues such as the liver, heart, and kidney (21, 44, 45). The effects of Li^+^ on the reproductive system through sperm mitochondria and consequent intracellular events, in two experimental models, has not been evaluated so far. Therefore, uncovering the mechanisms of Li^+^ adverse effects in the reproductive system could lead to the development of therapeutic strategies against xenobiotics-induced reproductive toxicity. Hence, the purpose of the current study was to uncover the intracellular events involved in Li^+^- induced male reprotoxicity through two different models (*in-vivo* and *in-vitro*).

In the current investigation, two experimental models were used. In the *in vitro* study, Leydig cells (LCs) were isolated from healthy mice, cultured, and exposed to increasing concentrations of Li^+^ (0, 5, 10, 50, and 100 ppm). In the *in vivo* experiments, male mice were treated with Li^+^ (10, 50, and 100 ppm, in drinking water) for 35 consecutive days. Serum, sperm, and testis tissue specimens were collected. Several indices, including biomarkers of OS, and mitochondrial functionality indices, as well as sperm parameters under various doses of Li^+^, were monitored.

## Materials and Methods

### Chemicals

2′,7′-Dichlorofluorescein diacetate (DCFH-DA), 3-[4,5dimethylthiazol-2-yl]-2,5-diphenyltetrazolium bromide (MTT), 4,2-Hydroxyethyl,1-piperazineethanesulfonic acid (HEPES), oxidized glutathione, dinitro fluoro benzene, bovine serum albumin, lithium chloride (Li^+^), 3-(N-morpholino) propane sulfonic acid (MOPS), ethylene glycol-bis (2-aminoethyl ether)-N, N, N′, N′-tetraacetic acid (EGTA), dimethyl sulfoxide, iodo-acetic acid, nigrosine, glutathione (GSH), malondialdehyde, potassium chloride, eosine, thiobarbituric acid, coomassie brilliant blue, sucrose, sodium chloride, dithiothreitol, rhodamine 123, and ethylenediaminetetraacetic acid (EDTA) were purchased from Sigma Chemical Co. (St. Louis, MO, USA). Trichloroacetic acid (TCA) and hydroxymethyl aminomethane hydrochloride (Tris-HCl) were purchased from Merck (Darmstadt, Germany).

### Experimental Setup

Thirty mature male Balb/c mice (25–30 g) were purchased from the Animal House of Shiraz Medical University, Shiraz, Iran. Controlled conditions (12:12 h, photoschedule; 18–22°C; appropriate ventilation, and 40% relative humidity) were set for experimental units. Animals had access to a commercial rodent pellet (RoyanFeed^®^, Esfahan, Iran) and tap water *Ad libitum*. All animal procedures were performed according to the guidelines of the ethics committee of Shiraz University of Medical Sciences, Shiraz, Iran (#97-01-36-16776). Mice were allotted to four trial *in-vivo* groups (*n* = 6 in each), as follow: (A) Control (vehicle-treated, 0 ppm lithium chloride in drinking water); (B) Li^+^ (10 ppm in drinking water); (C) Li^+^ (50 ppm in drinking water); (D) Li^+^ (100 ppm in drinking water). Lithium chloride was treated daily for 35 consecutive days (*in-vivo)* and 48 h (*in-vitro*). After the trial exposure time, animals were anesthetized (thiopental 80 mg/kg, i.p), and samples were collected. A wide range of Li^+^ concentrations were used in the current study (10–100 ppm). These concentrations are closed to plasma level of these drugs in patients (e.g., bipolar patients) (46). The toxicity of Li^+^ in animal models is also in the range of Li^+^ concentrations used in the current study (47, 48).

According to the procedure described in previous studies (49, 50), Leydig cells (LCs) were isolated from dissected testes. Afterward, the isolated LCs were added to 24-well (2 × 10^5^ cells/mL/well) plates containing Ham's F12/DMEM culture medium supplemented with 10% FBS and 1% myllicin. All *in-vitro* culture methods took place in a 37°C controlled humidified conditions (5% CO_2_ in the air). The isolated cells were incubated for 2 days. Then, cells were washed twice with a serum-free medium. Afterward, the washed LCs were challenged with different doses of Li, similar to that exposed to *in-vivo* study (0, 10, 50, and 100 ppm) for 48 h.

### Sample Collection, LCs Isolation, and Evaluation

Animals were deeply anesthetized with thiopental (80 mg/kg, i.p). The epididymides and testes were excised and weighed. The left testes were kept in a buffered formalin solution (10% formalin in phosphate buffer, pH = 7.4) for histopathological assessments. Total antioxidant capacity, lipid peroxidation, reactive oxygen species (ROS) production, protein carbonylation, and glutathione contents were determined in the right testes, spermatozoa, and LCs. Sperm sample was collected from the left cauda epididymis.

For evaluating the effects of Li^+^ on LCs, these cells were isolated from healthy mice and cultured (50). For LCs isolation male gonads were quickly removed and dissected. The *tunica albuginea* and all three parts of epididymides were gently removed to expose the seminiferous tubules (ST). Then, each dissected gonad was transferred into a sterile plastic plate containing 2.5 ml phosphate-buffered saline (PBS; pH = 7.4). The testis was chopped with a tweezer in the shaking water bath (37°C) until the PBS became opaque. Subsequently, the isolated cells were centrifuged (200 *g*, 5 min), the supernatant was discarded and the pellet was resuspended in the F12/DMEM medium (4.5 ml, Gibco, NY, USA) supplemented with fetal calve serum (FBS; 10%, Gibco, NY, USA), 100 μg/mL streptomycin, and 100 U/mL penicillin in a standard incubation condition (37°C, 5% CO_2_). Giemsa staining was applied for morphological identification of cultured LCs.

### Bodyweight Gain, and Organ Weight Index

Mice were weighed at the first and final days of the trial, and the bodyweight gain in each group was recorded. Testicular, epididymal, and vas-deferens weight indices were determined as weight index = [wet weight of organ (g)/body weight (g)] × 100.

### Sperm Indices

The eosin-nigrosin staining was used to evaluate spermatozoa viability and abnormality (51, 52). Briefly, the extracted caudal epididymal germ cells (100 μL) were incubated in PBS (35°C; pH = 7.4). Spermatozoa smear was stained with eosin-nigrosin (10 μL). Slides were monitored randomly for viability assay, where unstained germ cells were considered live (51, 52). The germ cells with ab-axial tail, protoplasmic droplets, double tails, malformed heads, coiled tails, bent tails, and without tail and head were considered abnormal. Spermatozoa concentration and progressive motility were also assessed based on previous studies (39, 41). Briefly, progressive motility of sperm was assessed by adding a drop of spermatozoa suspension on a glass slide covered with a coverslip. Samples were observed under a light microscope (400 × magnification, Zeiss, Jena, Germany) equipped with hot-stage (35°C). Hypoosmotic swelling test (HOST) was applied to assess the integrity of the spermatozoa plasma membrane. Briefly, 10 μL of germ cells were suspended in 50 μL of a hypo-osmotic solution (50-mOsm NaCl). Then, samples were then incubated for 10 min at 37°C. Finally, the spermatozoa percentage with a swollen “bubble” around the curled flagellum through counting 200 germ cells, were randomly counted on each slide using a light microscope (1,000 × magnification) (53, 54). The concentration of germ cells was assessed by a Neubauer hemocytometer using light microscopy (200 × magnification).

### Oxidative Stress Indices in Testis, Sperm, and Cultured LCs

#### Reactive Oxygen Species (ROS)

Testicular, spermatozoa, and cultured LCs' ROS level was estimated using dichlorofluorescein diacetate (DCFH-DA) (55–59). Briefly, DCFH-DA was added (10 μM final concentration) to sperm (10^6^ sperm/mL), isolated LCs (10^4^ cells/well), and homogenized testicular samples (1 mg protein/mL). Specimens were incubated in the dark (15 min, 35°C). Afterward, the DCF fluorescence intensity was measured using a FLUOstar Omega^®^ fluorimeter (BMG LABTECH^®^, Germany) at λ _excitation_ = 485 nm and λ _emission_ = 525 nm (37, 58, 60).

#### Lipid Peroxidation

Thiobarbituric acid reactive substances (TBARS) test was used to assess lipid peroxidation in the current study (61–63). For this purpose, 500 mg of testis tissue homogenate (10% w/v in KCl, 1.15% w: v), samples of 10^6^ sperm/mL, and isolated LCs (10^4^ cells/well) were added to a reaction mixture consisting of 1 mL thiobarbituric acid (0.375%, w: v) and 1 mL phosphoric acid (1% w: v, pH = 2) (63–66). Then, samples were heated in a water bath (20 min, 100°C). Afterward, 1 mL of n-butanol was mixed and mixed. Samples were centrifuged (10,000 *g*, 5 min) and the absorbance of the the n-butanol phase (upper phase) was measured (λ = 532 nm, EPOCH^®^ plate reader, USA) (66–68).

#### Glutathione Content of the Testis Tissue, LCs, and Sperm Samples

An HPLC apparatus with an NH_2_ column (25 cm; Bischoff chromatography, Leonberg, Germany), was used to determine nanomole levels of reduced (GSH) and oxidized (GSSG) glutathione in deproteinized specimens (39, 69). Water and acetate (Buffers A; 4:1 v: v) along with a mixture of methanol with buffer A (Buffers B; 4:1 v: v) were considered as the mobile phases (flow rate = 1 mL/min). A gradient technique with a constant upsurge of buffer B to 95% in 25 min was applied (70). Samples (500 μL of testis homogenate) was treated with 50 μL of TCA (50% w: v, 4°C, in MiliQ water). Sperm (1 mL of 10 ^6^ sperm/mL) and LCs (1 mL of 10 ^6^ cells/mL) were also treated with 100 μl of ice-cooled TCA (50% w: v, 4°C). Samples were incubated on ice (30 min). After the incubation period, samples were mixed well and centrifuged (17,000 g, 15 min, 4°C). Afterward, the supernatant was added to 5 mL tubes and treated with 0.5 mL of NaOH: NaHCO_3_ solution (2 M: 2 M). Then, 0.1 mL of iodoacetic acid (1.5% w: v in MiliQ water) was added and the mixture was incubated in the dark (60 min; 4°C). Afterward, 0.5 mL of DNFB (1.5% v: v in HPLC grade ethanol) was carefully mixed and incubated in the dark (25°C, 24 h). Samples were centrifuged (17,000 *g*, 30 min, 4°C) and filtered. Finally, 25 μL of each specimen was injected into the HPLC apparatus. The UV detector was set at λ = 252 nm (71).

#### Ferric Reducing Antioxidant Power (FRAP)

Total antioxidant capacity of testicular tissue, LCs, and sperm specimens was measured using the FRAP assay (72–74). Briefly, the working FRAP mixture was freshly prepared by mixing 25 mL of acetate buffer (300 mmol/L, pH = 3.6), 2.5 mL of TPTZ (2, 4, 6-tripyridyl-s-triazine, 10 mmol/L in 40 mmol/L hydrochloric acid), and 2.5 mL of ferric chloride (20 mmol/L). Testicular, LCs, and sperm specimens were homogenized, separately, in 0.25 M of Tris-HCl buffer (pH = 7.4; 4°C), containing 0.2 M sucrose and 5 mM dithiothreitol (DTT) (75, 76). Afterward, FRAP reagent (1.5 mL) and deionized water (150 μL) were added to 100 μL of homogenates and incubated at 37°C (5 min, in the dark). Finally, the absorbance was measured at λ = 593 nm (EPOCH plate reader, USA) (72, 77–79).

#### Protein Carbonylation

The testicular and cultured LC's protein oxidative damage of Li- exposed groups, was assessed by the determination of carbonyl groups based on the reaction with dinitrophenylhydrazine (DNPH) (80, 81). In summary, the tissue homogenate (1,000 μL, 10% in KCl) and cultured LCs specimens (1 mL; 10^4^ cells/well) were mixed with 0.1 mL of TCA (20% w: v, 4°C) and centrifuged (700 *g*, 5 min, 4°C). Then, the supernatant was mixed with 0.3 mL of 2, 4-dinitrophenylhydrazine (DNPH; 10 mM, dissolved in 2 M HCl). Samples were incubated at room temperature (25°C, 60 min, in the dark, regularly vortexed every 10 min). Then 0.1 mL of trichloroacetic acid (20% w: v, 4°C) was added, and centrifuged (12,000 *g*, 10 min). The pellet was washed (three times) with 1 mL of ethanol: ethyl acetate (1:1 v: v). The final pellet was dissolved in 0.6 mL of guanidine solution (6 M, in 20 mM potassium phosphate, pH = 2.3). Samples were centrifuged (12,000 *g*, 5 min), and the absorbance of the supernatant was measured at λ =370 nm (EPOCH plate reader, USA) (72).

### Measuring Mitochondrial Indices in Mice Epididymal Spermatozoa, and Cultured Lcs

#### Mitochondrial Dehydrogenase Activity

The 3-(4, 5-dimethylthiazol-2-yl)-2, 5-diphenyltetrazolium bromide colorimetric method was used for the assessment of mitochondrial dehydrogenases activity in sperm and isolated LCs. For this purpose, the specimens (1 mg protein/mL) were incubated at 37°C for 30 min (in the dark) with 40 μL MTT solution (5 mg/mL). Samples were centrifuged and the pellet was dissolved in 1,000 μL of DMSO. The absorbance was measured at λ = 570 nm using the EPOCH^®^ plate reader (63, 82–84).

#### Mitochondrial Depolarization

Mitochondrial uptake of rhodamine-123 was applied as a method for the determination of sperm and cultured LCs mitochondrial depolarization (55, 80, 83, 85, 86). Briefly, the specimens (1 mg protein/mL) were incubated with rhodamine-123 (35°C, 15 min, in the dark) and then centrifuged (10,000 *g*, 5 min, 4°C) (87–89). Finally, the fluorescence intensity (FI) of the supernatant was measured at λ _excitation_= 485 nm and λ _emission_ = 525 nm using a fluorimeter (BMG LABTECH^®^, Germany) (55, 83, 90).

#### Assessment of ATP Levels

The ATP level in sperm and LCs was assessed by an HPLC method (91). For this purpose, LCs (10^4^ Cells/mL) and sperm specimens (10^6^ sperm/mL) were treated with 100 μL ice-cooled meta-phosphoric acid (50% w: v, 4°C). Samples were mixed and incubated on ice for 10 min. Afterward, samples were centrifuged (30 min, 17,000 *g*, 4°C), and the supernatant (100 μL) was treated with ice-cooled KOH (10 μL of 1 M solution) (92). Samples were centrifuged (30 min, 17,000 *g*, 4°C), and 20 μL of the supernatant was injected into an HPLC system. The HPLC system consisted of an LC-18 column (25 cm μ-Bondapak^®^ column). The mobile phase was a mixture of potassium phosphate buffer (100 mM KH_2_PO_4_, pH = 7 adjusted with KOH), tetrabutylammonium hydroxide (1 mM), and acetonitrile (2.5% v: v) (63, 89, 93). An isocratic method with the flow rate was 1 mL/min was used. The UV detector was set at λ = 254 nm (63, 94).

#### Testosterone (T) Biosynthesis in Li^+^-Exposed LCs

An ELISA-based testosterone assay kit (Elabscience, Wuhan, China) was used to measure T content in Li^+^-exposed LCs [44]. For T generation, isolated LCs (2 × 10^5^ cells/well) were seeded in 24-well plates. After 24 h of incubation, the quality of cells was monitored and then cultured with various doses of Li^+^ (0, 10, 50, and 100 ppm) for 48 h. After the exposure time, media were gathered, and centrifuged (3,000 rpm, 10 min). The supernatant was used for assessing T levels.

#### Cellular Lactate Dehydrogenase (LDH) Release

A commercial kit (Pars Azmun^®^, Tehran, Iran) and Mindray^®^ BS-200 autoanalyzer (Guangzhou, China) were used to assess cells lactate dehydrogenase (LDH) levels in cultured LCs (95, 96). Isolated LCs were treated with different concentrations of Li^+^ (0, 10, 50, and 100 ppm) after the incubation period. Afterward, 200 μL of the culture medium was taken, and the LDH content was measured.

#### LCs Viability Assay

The viability of LCs was examined based on a colorimetric technique using MTT (49). Briefly, the LCs were seeded in quadruplicate into 96-well plates at a density of 1 × 10^4^ cells/well and allowed to attach into the cells overnight at 37°C with 5% CO_2_. LCs were challenged with Li^+^ (0, 10, 50, and 100 ppm) for 48 h at 37°C. Afterwards, the LCs (1 × 10 ^4^ cells/well) were incubated with MTT (0.4%, 37°C, 30 min, 5% CO_2_). Plates were centrifuged (3,000 g, 15 min) and the purple pellet was dissolved in l,000 μL dimethyl sulfoxide (DMSO). Finally, the optical density (OD) was measured at λ = 570 nm (EPOCH plate reader, BioTek^®^ Instruments, USA) (21, 97).

#### Testicular Histopathological Alterations

Male gonads were fixed in a buffered formalin solution (10% formaldehyde in phosphate buffer; pH = 7.4). The fixed tissue was paraffin-embedded and specimens were sectioned (5 μm) using a rotary microtome. Tissue sections were then stained with hematoxylin-eosin (H&E). Testicular histopathological changes were monitored and recorded by a specialized pathologist in a blind manner using a light microscope (Olympus BX41; Olympus Optical Co. Ltd, Japan). Testis tissue histopathological alterations were quantified as previously reported (98–100).

### Statistical Analysis

Data were expressed as Mean ± SEM. Data analysis was performed by the one-way analysis of variance (ANOVA), and mean comparison performed using the Tukey's multiple comparison test as the *post hoc* test at *P* < 0.05 (GraphPad Prism 8).

## Results

### Bodyweight Gain and Organ Weight Index

**Figure 1:**
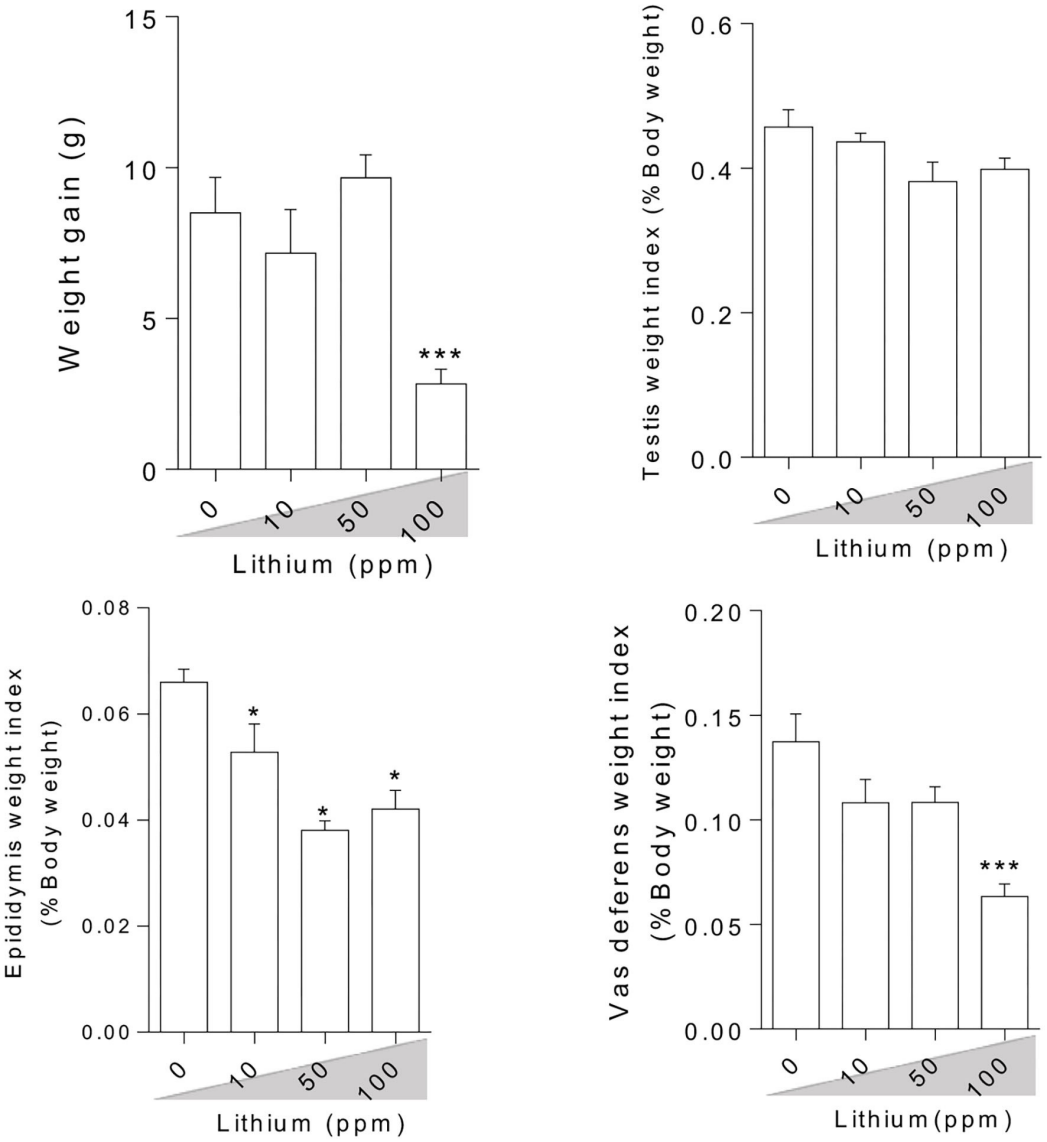
Effect of lithium on animals' weight gain and male reproductive organ weight index. Data are presented as mean ± SEM (*n* = 6). Asterisks indicate significantly different from the control (0 ppm) group (^*^P < 0.05, ^***^P < 0.001).

Bodyweight and reproductive organ weight index measurement in the Li^+^-exposed mice uncovered significant variations (Figure 1). It was observed that live bodyweight considerably decreased in the group receiving a high dose of Li^+^ (100 ppm). On the other hand, the weight index was significantly reduced in the epididymis weight index of the mice exposed to various doses of Li^+^ (10, 50, and 100 ppm) and vas-deferens weight index of the animal challenged with the highest dose of Li^+^ (100 ppm). However, there was no significant difference in the testis weight index among all trial groups (Figure 1).

### Sperm Indices

**Figure 2:**
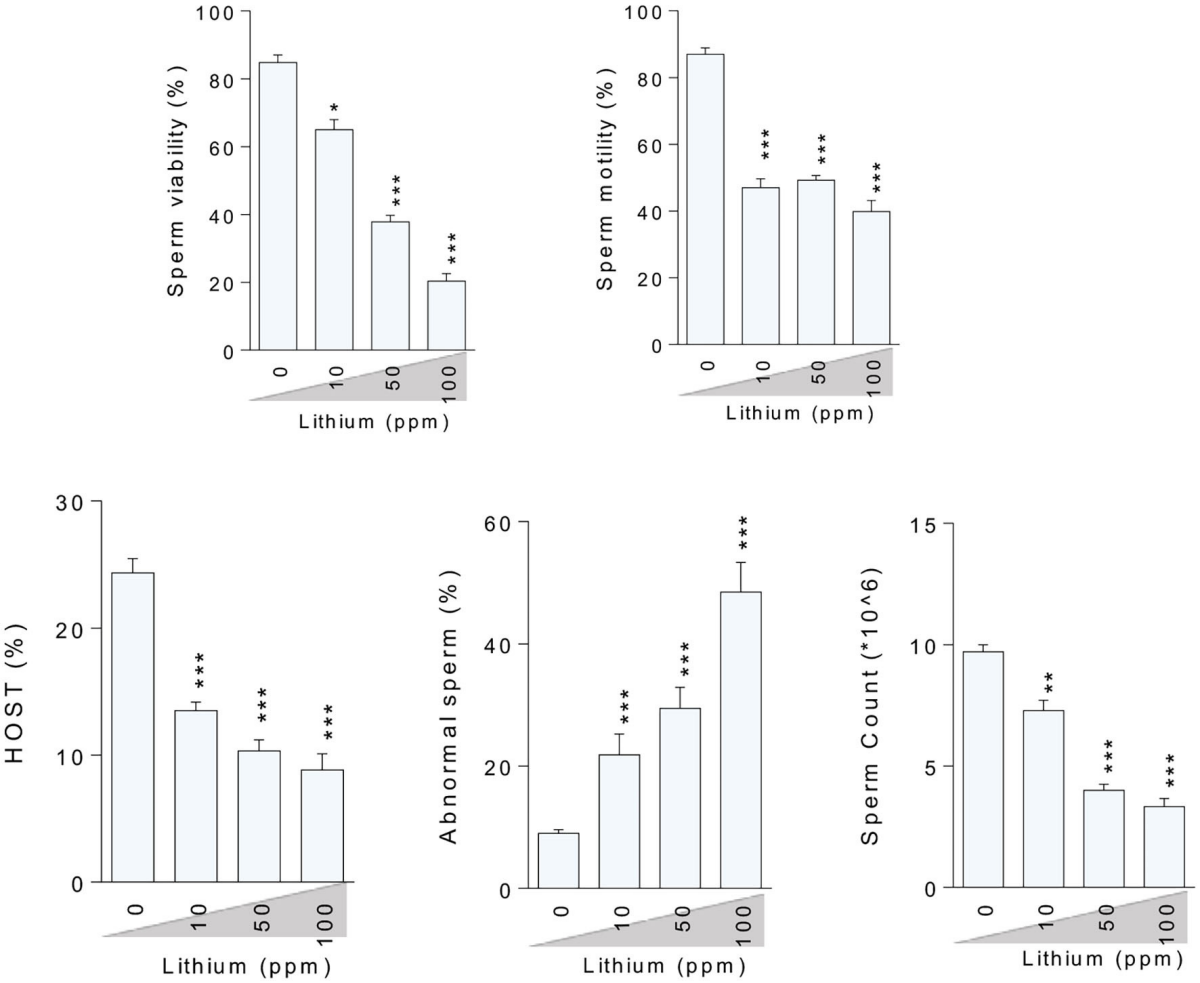
Effect of lithium administration on epididymal sperm parameters in mice. HOST: Hypo-osmotic swelling test. Data are presented as mean ± SEM (*n* = 6). Asterisks indicate significantly different from the control (0 ppm) group (^*^P < 0.05, ^**^P < 0.01, ^***^P < 0.001).

Assessment of the epididymal spermatozoa indices showed a dose-dependent increment in the percentage of abnormality along with a decrement in the rate of spermatozoa viability, motility, HOST, and sperm count in the Li^+^- challenged mice, in a dose-dependent manner (except of the motility index) (Figure 2).

### Oxidative Stress Biomarkers

**Figure 3:**
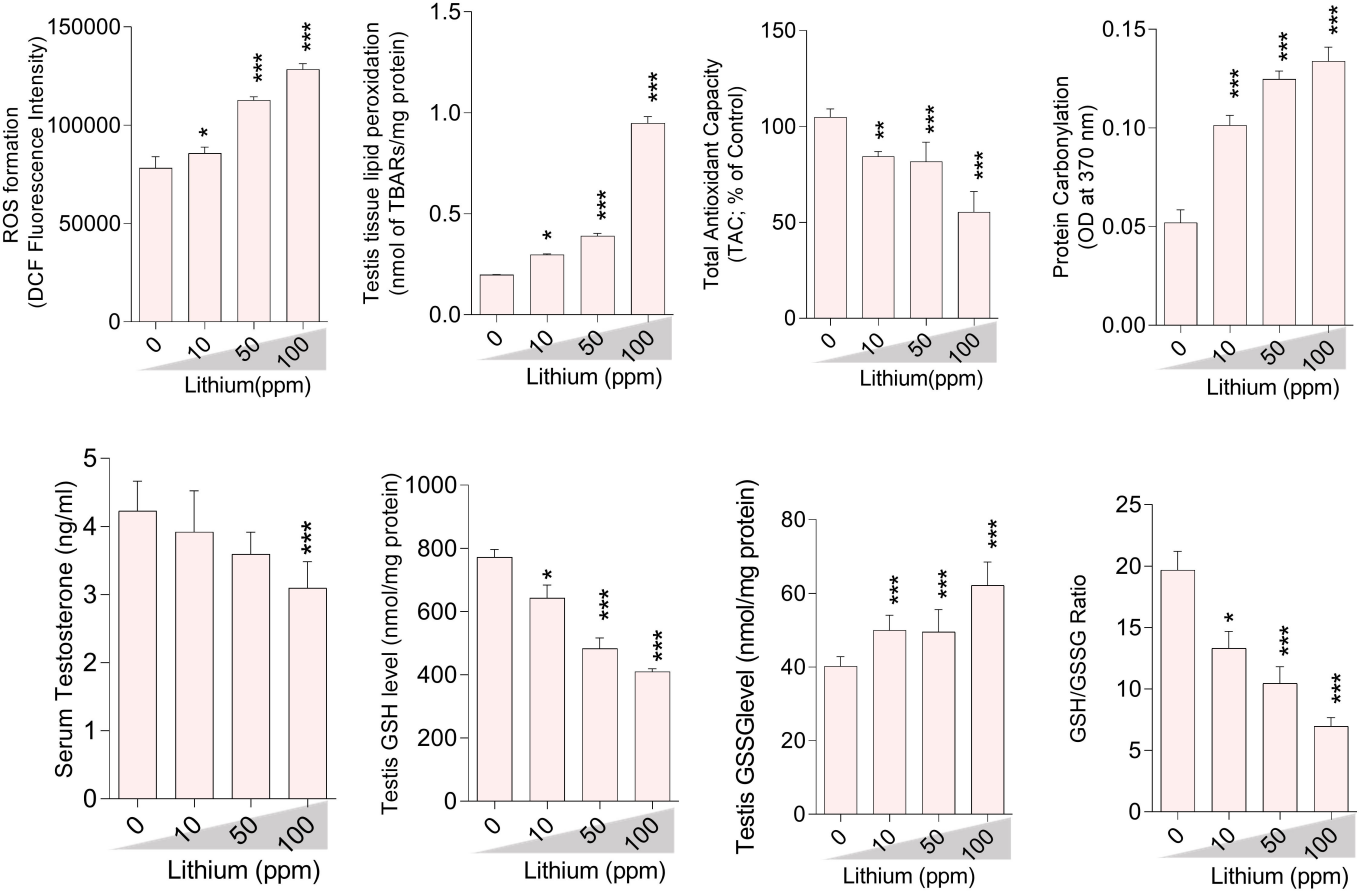
Biomarkers of oxidative stress in the testis tissue of lithium-treated mice. Data are presented as mean ± SEM (*n* = 6). Asterisks indicate significantly different from the control (0 ppm) group (^*^P < 0.05, ^***^P < 0.001).

**Figure 4:**
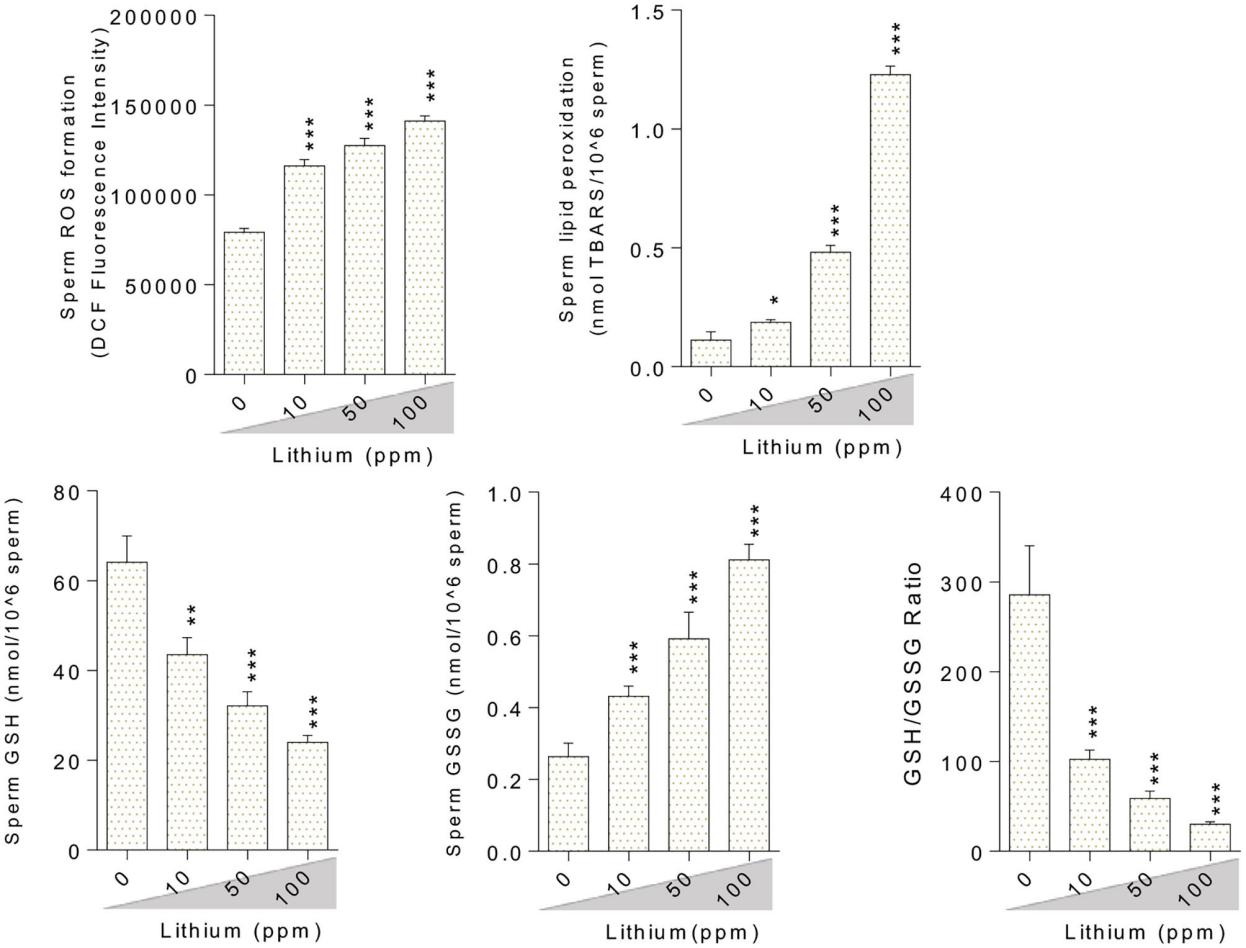
Biomarkers of oxidative stress in the epididymal sperm of lithium-exposed mice. Data are presented as mean ± SEM (*n* = 6). Asterisks indicate significantly different from the control (0 ppm) group (^*^P < 0.05, ^**^P < 0.01, ^***^P < 0.001).

**Figure 5:**
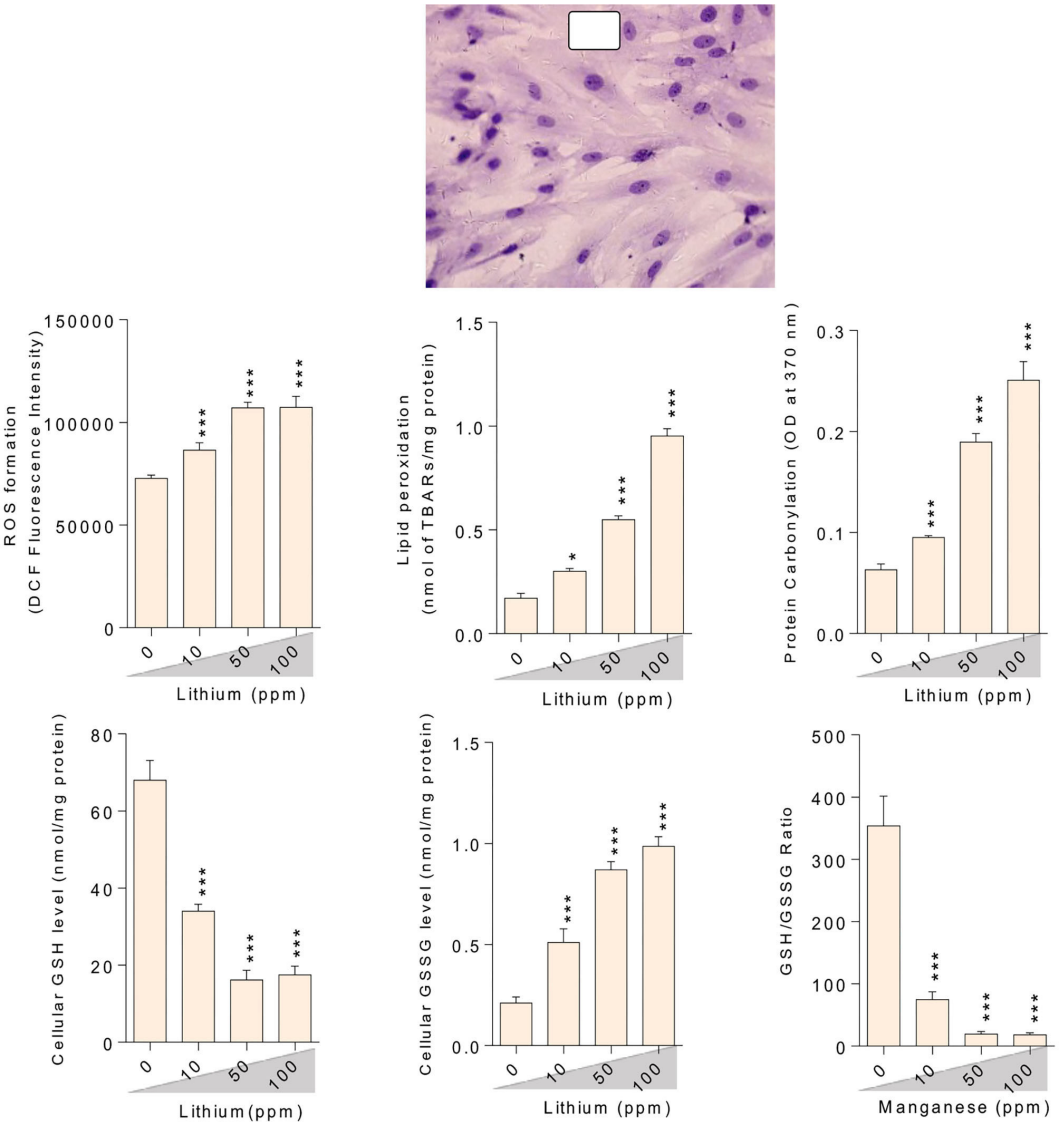
Lithium-induced mitochondrial impairment in mice epididymal germ cells. Data are presented as mean ± SEM (*n* = 6). Asterisks indicate significantly different from the control (0 ppm) group (^*^P < 0.05, ^***^P < 0.001).

Oxidative stress indices were assessed in the testis tissue (Figure 3), epididymal spermatozoa (Figure 4) of the male mice (*in-vivo*), and isolated/cultured LCs (*in-vitro)* challenged with various doses of Li^+^ (Figure 5; Giemsa staining was used for morphological observation of cultured LCs). Significant ROS levels in addition to lipid peroxidation, protein carbonylation, and oxidized glutathione (GSSG) were found in the testis tissue, extracted epididymal spermatozoa, and cultured/exposed LCs in Li^+^-treated groups, approximately in a dose-dependent-manner (Figures 3–5). Meanwhile, it was also observed that total antioxidant capacity (TAC) along with glutathione (GSH) level and the ratio of GSH / GSSG were significantly decreased in Li^+^-exposed groups (Figures 3–5).

### Mitochondrial Indices, Cytotoxicity Markers, and Testosterone Biosynthesis

**Figure 6:**
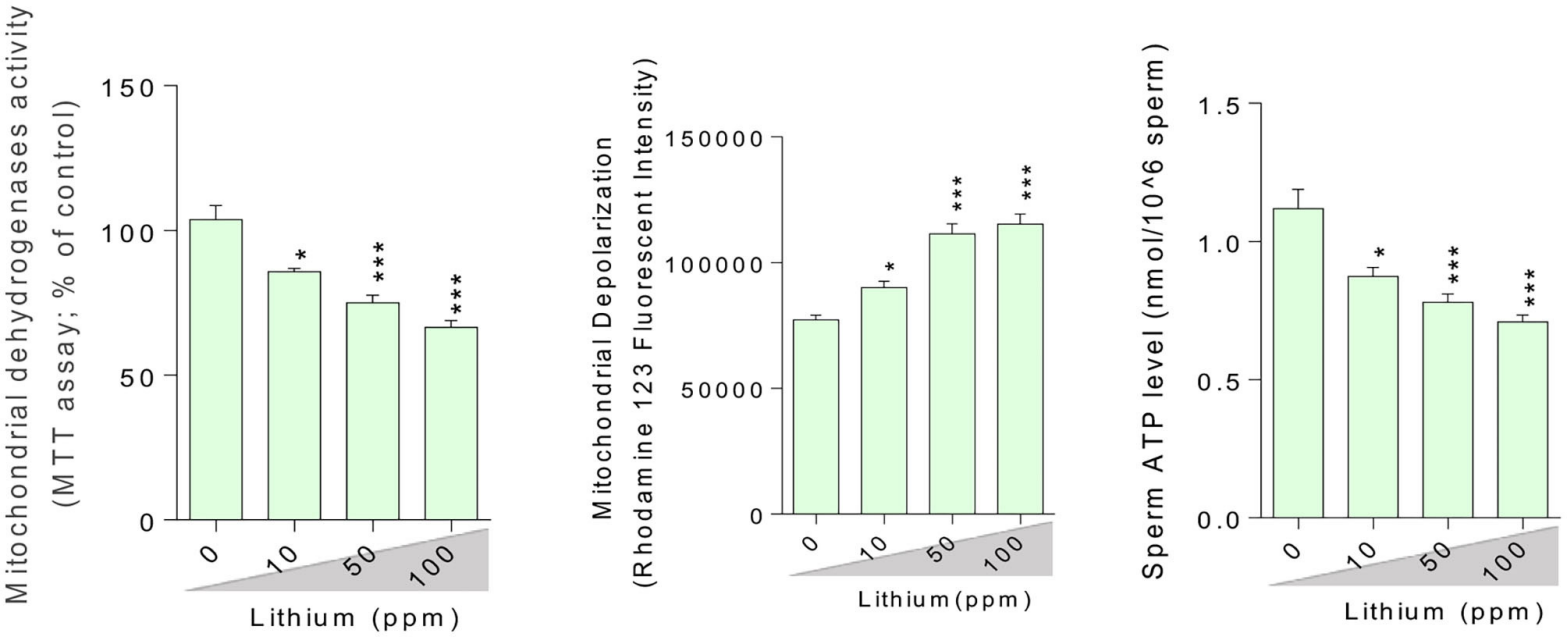
Light micrograph of testis tissue histopathological alterations in normal and lithium-treated mice. H&E staining (400× magnification). The grades of histopathological changes are given in Table 1. A severely degeneration and altered seminiferous tubule (A), and a massive loss of elongated spermatozoa (¤**)**, as well as a disappearance and degeneration of interstitial cells (LCs) from the interstitial space (^*^) was recorded.

**Figure 7:**
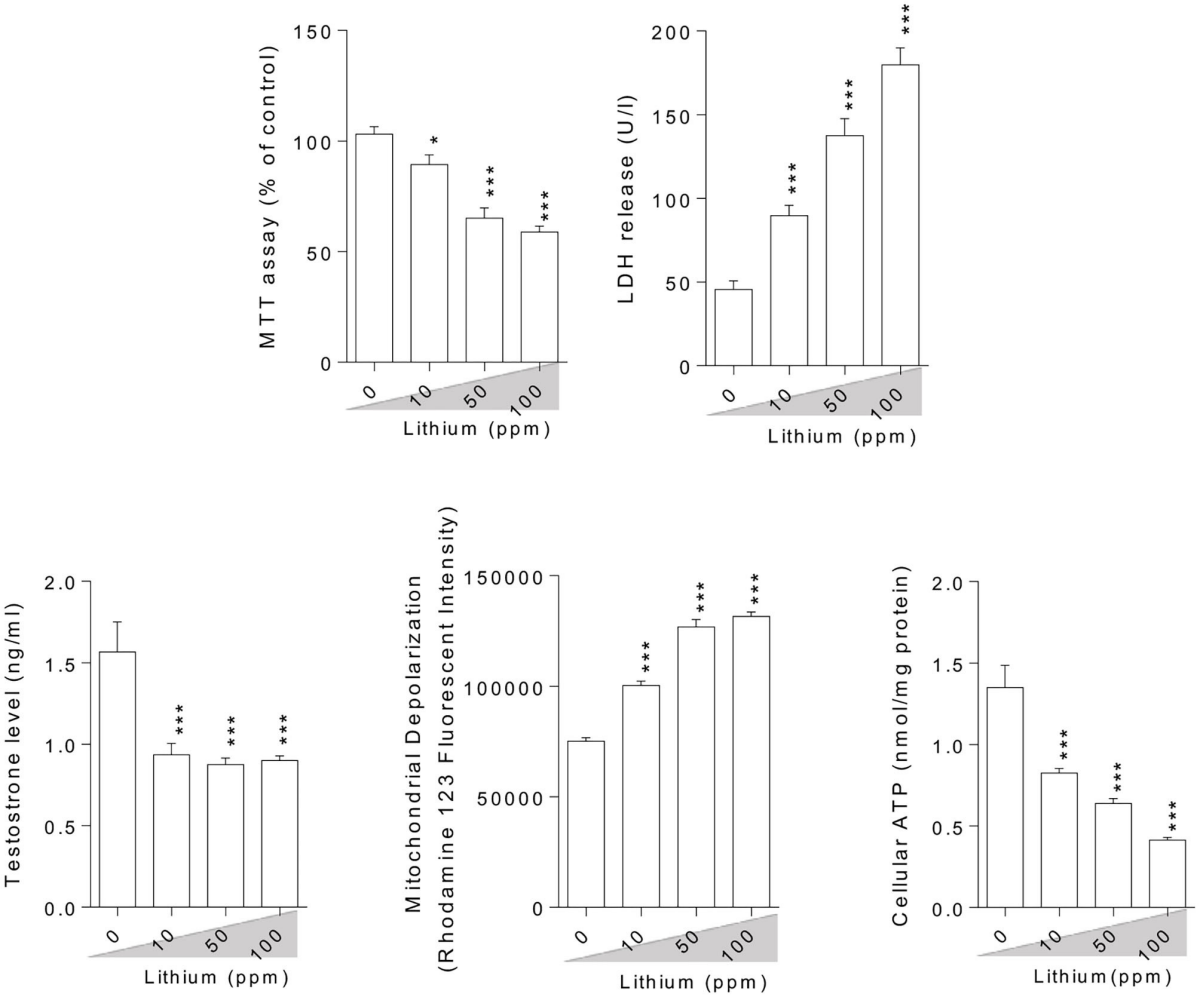
Effect of lithium exposure on biomarkers of oxidative stress in Leydig cells (LCs) isolated from mice testis. A: Giemsa staining (magnification at × 400) of cultured LCs. Data are presented as mean ± SEM (*n* = 6). ^*^ & ^***^ Significantly different from the control group (*P* < 0.05 and *P* < 0.001, respectively).

Epididymal spermatozoa and cultured/exposed LCs mitochondrial parameters revealed significant mitochondrial depolarization, decreased dehydrogenases activity, and depleted ATP content in Li^+^-exposed groups, in a dose-proportional manner (Figures 6, 7). Along with these alterations, our data showed that cellular LDH release and T levels were dose-dependently increased and significantly decreased in mice isolated/exposed LCs, respectively (Figure 7). Serum testosterone levels were also significantly lower than the control group in mice exposed to 100 ppm of Li^+^ (Figure 3).

### Histopathology Alterations of the Testis Tissue

**Figure 8:**
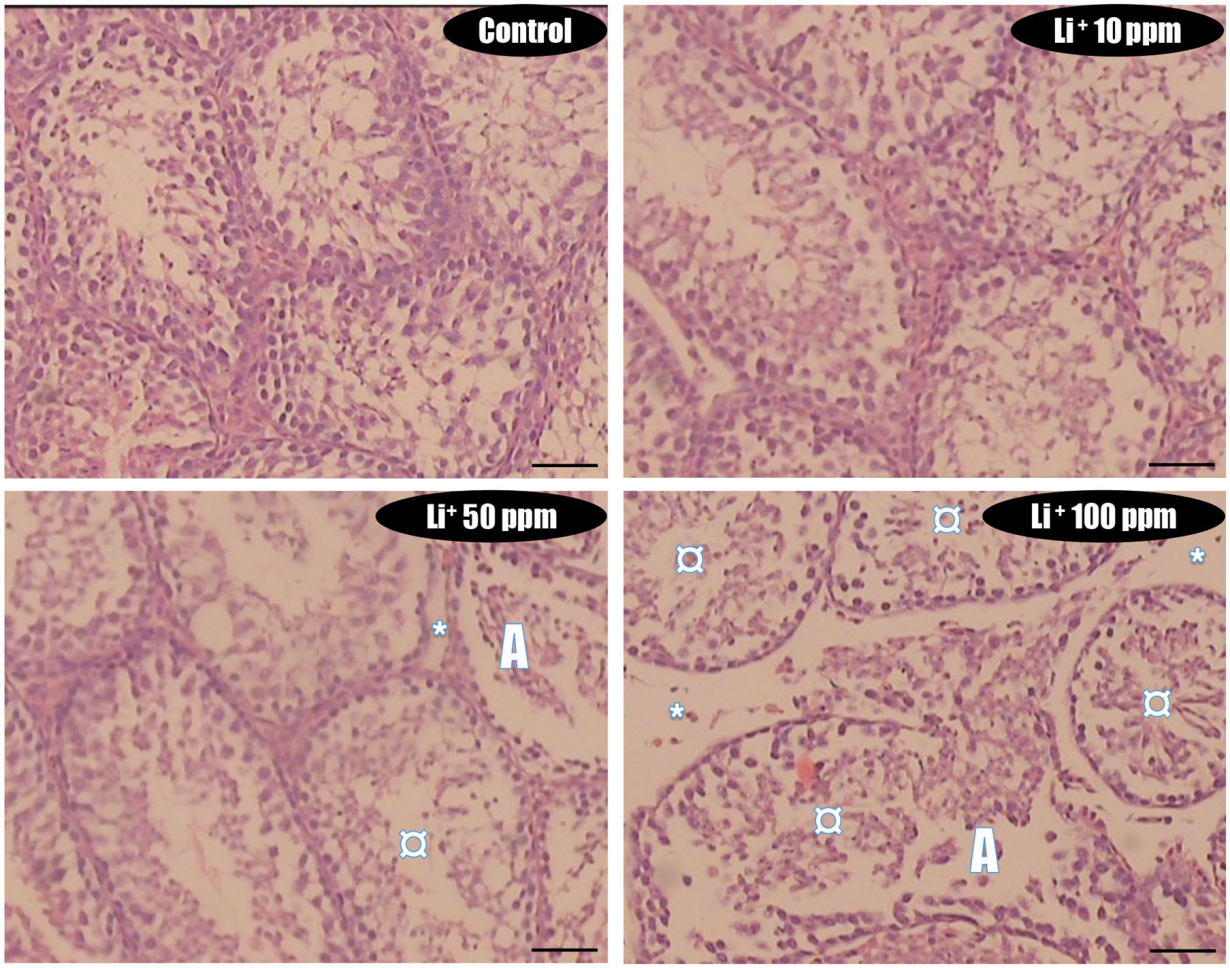
Lithium-induced cytotoxicity and mitochondrial impairment in mice Leydig cells. Data are presented as mean ± SEM (*n* = 6). ^*^ & ^***^ Significantly different from the control group (*P* < 0.05 & *P* < 0.001, respectively).

**Figure 9:**
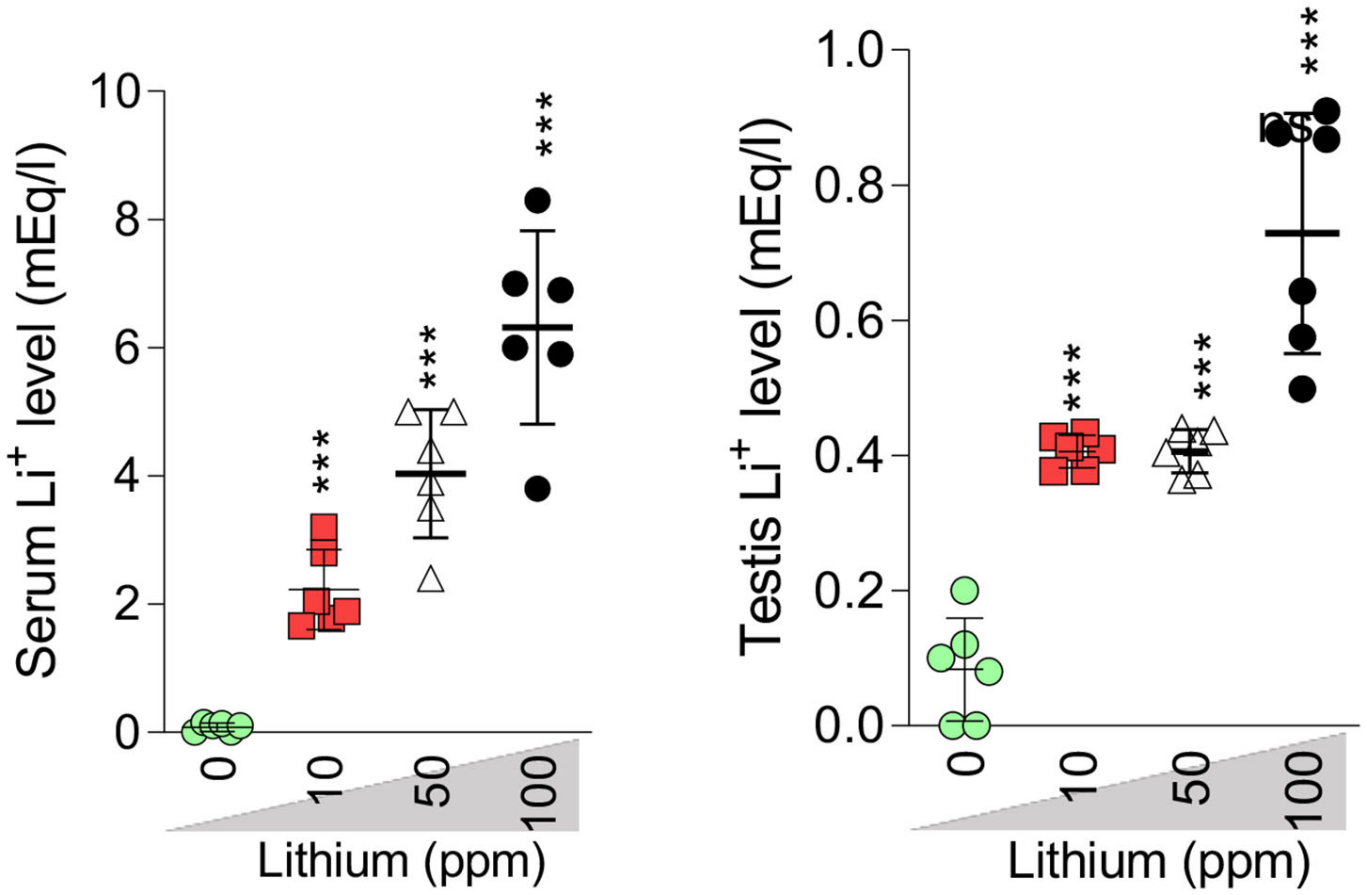
Serum and testis level of lithium (Li^+^). Data are represented as mean ± SEM (*n* = 6). ^***^ Indicate significantly different as compared with the control (Li^+^ 0 ppm) (*P* < 0.001).

In Li^+^-exposed mice with various doses (10, 50, and 100 ppm in drinking water/day), the male gonad revealed the severe tubular injury, tubular desquamation, and low spermatogenic index. However, the maximum alterations were observed in the group received the highest dose (100 ppm) of Li^+^ (Table 1 and Figure 8). No significant histopathological changes were detected between the groups of low (10 ppm) and medium (50 ppm) (Figure 8). It is noteworthy that serum and testis tissue levels of Li^+^ was significantly higher in drug-treated groups (Figure 9).

**Table 1:** Testis tissue histopathological alterations in lithium-exposed mice.

	**Tubular injury**	**Tubular desquamation**	**Spermatogenic index**
Control (Lithium 0 ppm)	–	–	1
Lithium 10 ppm	+	++	1
Lithium 50 ppm	++	+++	0.8
Lithium 100 ppm	+++	+++	0.75

*+: Mild; ++: Moderate; and +++: Severe histopathological alterations*.

## Discussion

Li^+^ is a drug used for the treatment of a wide range of neurological disorders (1). Despite its tremendous clinical value, several adverse effects have been attributed to Li^+^ (1). Several reports of Li^+^-induced reproductive toxicity have also been reported (2, 16, 20, 26). Unfortunately, there is no precise mechanism(s) for Li^+^-induced reproductive toxicity. Hence, this investigation was planned to monitor the deleterious effects of Li^+^ on reproductive indices of male mice in two *in-vitro* and *in-vivo* models. It was found that Li^+^-induced mitochondrial impairment and oxidative stress play a significant role in its adverse effects on male reproductive system.

Several investigations mentioned the primary role of oxidative stress (OS) in the pathogenesis of reproductive system dysfunction (16, 20, 26, 101). Many anomalies in the functionality of spermatozoa are attributed to the increased level of ROS in testis and semen (37–40) through severe impairments of DNA, interruption of plasma membrane integrity, and denaturation of proteins (41–43). It is well–known that interstitial cells (Leydig cells; LCs) into the male gonad are formed of many lipid-filled vesicles; therefore, the LCs are liable to OS via the exogenous ROS producing by mitochondria as their pivotal by-products (50). Quinn and Payne (102) have also been shown that steroidogenic- related cytochrome p450 enzymes can produce ROS in the LCs through the mechanisms related to their catalytic reactions.

There is no precise mechanism for the source of ROS in males treated with Li^+^. On the other hand, there is no specific description for reproductive indices abnormalities (e.g., sperm motility) in Li^+^-treated patients. In line with previous studies, we found that OS is involved in the mechanism of testis and sperm injury in Li^+^-treated animals. The current study also revealed the effects of Li^+^ on testosterone biosynthesis in both *in vivo* and *in vitro* models. More importantly, we found that mitochondrial impairment could play a critical role in sperm abnormality, LC's injury, and testis injury in Li^+^-exposed groups. Li^+^-induced mitochondrial impairment and decreased ATP levels could induce decreased sperm motility.

Finding the mechanism(s) of xenobiotics-induced organ injury is a critical step in developing therapeutic options. Li^+^ is a drug with adverse effects on the reproductive system. There are several lines of evidence indicating the pivotal role of OS in the pathogenesis of Li^+^-induced reproductive toxicity (2, 16, 20, 26). At the sub-cellular levels, we found that sperm mitochondria are a critical target for Li^+^-induced sperm abnormalities. The motility of sperm in Li^+^-treated groups was significantly lower as compared with the control animals (Figure 2). This important data could indicate the lack of energy (ATP) for sperm motility. Moreover, we also found that LC's ATP level was significantly depleted. This event could hamper the physiological role of these basic testes' cells in the synthesis and secretion of testosterone (Figure 7). All these events could finally lead to reduced reproductive performance in patients who are treated with Li^+^ for a long time.

Disturbed sperm mitochondrial function in Li^+^-treated animals could occur through several pathways. It has been found that Li^+^ can readily enter the mitochondrial matrix. Li^+^ could disrupt mitochondrial membrane potential as the driving force for ATP production (44, 45, 103). It has also been found that Li^+^ could adversely interact with mitochondrial electron transport chain (44, 45, 103). Li^+^ is also able to increase mitochondrial permeability (mPT) and ease the release of various cell death mediators to the cytoplasm (44, 45, 103). All these events could finally lead to cell death.

In the current study, we found that Li^+^ administration significantly impaired sperm parameters in the drug-treated mice. The adverse effects of this drug on mitochondrial function and OS parameters seems to play a pivotal role in its adverse impact on the reproductive system. On the other hand, we found that the treatment of isolated LCs with Li^+^ caused a significant decrease in testosterone production, increased LDH release, and a substantial reduction in cell viability (MTT assay) (Figure 7). Moreover, mitochondrial membrane potential and ATP levels were significantly decreased in Li^+^-exposed LCs (Figure 7). Mitochondrial impairment could lead to an energy crisis. As the proper mitochondrial function is a critical factor for proper sperm function (e.g., sperm motility) (104), any changes in mitochondrial function could lead to sperm abnormalities. Altogether, all these data indicate a role for OS and mitochondrial impairment in the pathogenesis of Li^+^-induced reproductive toxicity. Hence, antioxidants and mitochondria protecting agents could serve as viable therapeutic options against this adverse effect. Recently, several antioxidant agents have been tested against Li^+^-induced organ injury (16, 105). On the other hand, we tested several safe and clinically applicable agents (amino acids and peptides), which could boost mitochondrial function and energy metabolism (34, 67, 106–109). We hope that these agents could finally find a clinical application against drug-induced organ injury (e.g., Li^+^-induced reproductive toxicity). Indeed, further studies are needed to identify the precise mechanisms of Li^+^-induced reproductive organ injury, the possible interaction of ancillary treatments with the pharmacological effects of Li^+^, and finally, translating these experimental data to the clinical settings.

## Data Availability Statement

The original contributions presented in the study are included in the article/supplementary material, further inquiries can be directed to the corresponding author/s.

## Ethics Statement

The animal study was reviewed and approved by All animal procedures were performed according to the regulations and guidelines of the local ethics committee at Shiraz University of Medical Sciences, Shiraz, Iran (#97-01-36-16776).

## Author Contributions

MO, RH, AJ, and HN conceived and planned the experiments. MAr, OF, ZJ, MN, KM, AA, MAz, and SR-M performed the measurements and collected data. MO, RH, AJ, and NA contributed to the interpretation and visualization of the results. SR-M, MO, RH, NA, AJ, and HN also revised the whole manuscript to prevent grammar and syntax errors. All authors contributed toward data analysis, drafting, revising the paper, gave final approval of the version to be published, and agreed to be accountable for all aspects of the work. ZJ was completely involved in the manuscript (data collection, draft preparation, data visualization, and the confirmation of the final draft).

## Conflict of Interest

The authors declare that the research was conducted in the absence of any commercial or financial relationships that could be construed as a potential conflict of interest.
